# Characterization of *Lactobacillus* spp. as Probiotic and Antidiabetic Potential Isolated from Boza, Traditional Fermented Beverage in Turkey

**DOI:** 10.1155/2024/2148676

**Published:** 2024-06-10

**Authors:** Chandana Kumari V. B., Sujay Huligere, Jayanthi M. K., Khang Wen Goh, Sudhanva M. Desai, Kalabharthi H. L., Ramith Ramu

**Affiliations:** ^1^Department of Biotechnology and Bioinformatics, JSS Academy of Higher Education and Research, Mysore 570015, Karnataka, India; ^2^Department of Pharmacology, JSS Medical College, JSS Academy of Higher Education and Research, Mysore 570015, Karnataka, India; ^3^Faculty of Data Science and Information Technology, INTI International University, Nilai 71800, Malaysia; ^4^Department of Chemical Engineering, Dayananda Sagar College of Engineering, Bengaluru, Karnataka, India

## Abstract

Boza, a cereal-based beverage popular in southeast Europe, is fortified with probiotics and is believed to positively impact the composition of the gut microflora. This investigation focused on fermented cereal-based beverage boza to identify strains of probiotic *Lactobacillus* spp. capable of inhibiting carbohydrate-hydrolysing enzymes *α*-glucosidase (AG) and *α*-amylase (AA). The isolated bacterial strains underwent a comprehensive assessment, including biochemical, molecular, and probiotic trait analyses such as tolerance survivability, adhesion, safety, and health-promoting attributes. We evaluated the inhibitory potential of the supernatant, cell lysate, and intact cells of *Lactobacillus* spp. Molecular analysis has revealed that isolates RAMULAB30 and RAMULAB29 exhibit a significant genetic similarity (>97%) to *Lacticaseibacillus paracasei* and *Limosilactobacillus fermentum*, respectively. These findings are documented in the NCBI database. They exhibited significant resistance to gastrointestinal and intestinal fluids, also indicating their potential for adhesion. Additionally, the isolates showed a significant antibacterial activity, particularly against *Micrococcus luteus*. They showed resistance to vancomycin and methicillin antibiotics but were more susceptible to streptomycin and ampicillin. Furthermore, the strains demonstrated antioxidant properties. To ensure their safety, a haemolytic assay was conducted despite their general recognition as safe (GRAS) status. The study primarily aimed to evaluate the inhibitory effects of the extract on enzymes AG and AA. Bacterial isolates demonstrated a significant inhibitory activity against both enzyme AG (32%–67% inhibition) and enzyme AA (18%–46% inhibition) in different forms, including supernatant (CS), lysed extract (CE), and intact cell (IC). These findings underscore the potential of bacterial isolates to inhibit the enzyme activity effectively. Furthermore, the *L. fermentum* RAMULAB29 and *L. paracasei* RAMULAB30 strains exhibit remarkable antidiabetic potential. Food products incorporating these strains have promising prospects as nutraceuticals, providing improved health benefits.

## 1. Introduction

The gut microbiota is now considered one of the most essential variables in host health control. The joint Food and Agriculture Organization (FAO) and World Health Organization (WHO) working group has officially defined probiotics as “live micro-organisms which, when administered in adequate amounts, confer a health benefit on the host” (FAO/WHO, 2001) [1]. Probiotics exert various effects, such as competition for nutrients, the production of antimicrobial compounds, the modulation of the immune system, and the anti-inflammatory potential [2]. In addition to human applications, probiotics are now being used to enhance the growth and health of farmed fish species [3]. *Saccharomyces boulardii* is a recognised probiotic with diverse therapeutic potential, particularly in the prevention and treatment of gastrointestinal disorders, including antibiotic-associated diarrhoea [4]. Furthermore, strains FX5 and FX9 of *W. paramesenteroides* isolated from fermented fruits exhibit promising probiotic properties and prebiotic utilisation abilities, making them suitable candidates for further investigation as synbiotics [5]. Numerous research works link variations in gastrointestinal microbiota with the development of diabetes [6, 7]. Around one quadrillion bacteria reside in the human gut, surpassing the total count of cells in the human body by about ten times [8]. Therefore, probiotics can be effective in managing diabetes and its consequences by altering the intestinal flora. Diabetes has received extensive consideration as a result of its high morbidity and mortality due to changes in dietary habits and lifestyle. Diabetes is a condition where carbohydrate uptake is improper, mainly caused by a lowered insulin secretion or no secretion at all [9, 10]. Targeting enzymes such as *α*-glucosidase (AG) and *α*-amylase (AA) may hold the key to effective medicinal intervention for reducing hyperglycemia [11]. The breakdown of complex oligo-/disaccharides into monosaccharides is facilitated by several enzymes, among which enzyme AA and enzyme AG enable absorption of the resulting monosaccharides through the intestinal wall [12, 13]. Although several drugs are available to treat diabetes, side effects and drug resistance are a major concern. A greater number of people are looking for natural products or dietary interventions to help prevent or treat diabetes [14]. LAB present in the gut and discovered from a wide number of different sources may have a key influence on various symptoms of diabetes [15, 16]. LAB, on the other hand, should be able to traverse and adhere to the digestive tract securely to have good benefits. Traditionally, LAB has been primarily isolated from milk and curd, which makes them widely used in the dairy industry [17–19].

Cereals are a staple cuisine all through the world. Rice, corn, maize, and wheat are the main staples of food and vary according to the availability of importance based on the places [20]. Fermentation is used to process a considerable amount of cereal harvest worldwide before it is consumed. It contributes to the taste, texture, and durability of food items while also supporting improved digestive processes. Cereal fermentation is a complicated process performed by the enzyme activity of microbes, which causes various biochemical reactions in raw materials [21, 22]. Probiotic LAB is an ideal candidate for fermentation from this standpoint as its consumption in adequate quantities can positively impact human health [14].

Boza is a typical Bulgarian fermented cereal-based beverage that undergoes a fermentation process, resulting in a unique flavour profile with pleasant sweet and sour notes reminiscent of bread. Boza is a fermented drink traditionally prepared using cereal as the primary ingredient that is popular in various regions of southeast Europe (Turkey, Romania, and Albania). LAB of the family of *Lactobacillaceae* (genus: *Lactobacillus* spp., *Pediococcus* spp., and *Leuconostoc* spp.) and the family *Streptococcaceae* (genus: *Lactococcus* spp.) can often ferment it [23, 24]. Boza can be made with components of millet (*P. miliaceum* and *P. sumatrense*), maize (*Z. mays*), oat (*A. sativa*), wheat (*Triticum L.*), and rice (*O. sativa*), as well as their mixtures. Wheat or bulgur wheat, a high-nutrient wheat product, can be made by crushing wheat and is mainly used in the preparation of boza. Vital amino acids, mineral reserves, and multivitamins are found in wheat, as well as its dietary fibre and beneficial phytochemicals, which are particularly abundant in whole grain products, which are important to the human diet [24, 25]. Tordorov et al. [24] have mentioned that boza is a natural potential resource for probiotic LAB and mainly *L. rhamnosus* spp. and *L. plantarum* spp. along with *L. pentosus* spp. and *L. paracasei* spp. were isolated from the boza sample. Earlier studies have investigated the antimicrobial potential of bacteriocin production by LAB strains that were isolated from boza [26, 27]. Our study represents a novel approach in the search for LAB with putative antidiabetic and probiotic traits. The main research aim of the present study was to identify and isolate LAB from the boza sample, which is known to possess potential probiotic properties and investigate their inhibition capability of carbohydrate-hydrolysing enzymes, specifically enzymes AG and AA.

## 2. Materials and Methods

### 2.1. Methodology for Identifying and Characterizing LAB

Cereal wheat was used in this investigation for the preparation of homemade boza, as described by Bayat and Yildiz [25]. Bulgur wheat and sugar were purchased locally from Mysore, Karnataka, India, and the process of boza was done as shown in Supplementary Figure S1 and using ingredients shown in Supplementary Table S1. The prepared sample was stored at 4°C. 1 mL of the sample was taken from the boza stock and serially diluted. The boza sample (serially diluted; 100 *μ*L) was seeded on *Lactobacillus* MRS agar (HiMedia Laboratories Pvt. Limited, India) (37°C, 24 h). The distinguishable colonies were chosen and screened for Gram-positive and catalase-negative strains. *Lactobacillus* MRS broth (HiMedia Laboratories Pvt. Limited, India) mixed with 15% (v/v) glycerol was used to preserve the pure cultures at −80°C [28]. Isolated cells (10^8^ CFU/mL) were prepared for the preliminary analysis (pH, temperature, salt, carbohydrate fermentation, and phenol), as per Kumari et al. [29].

### 2.2. Genetic Characterization and Phylogenetic Analysis of LAB

The isolated LAB strains underwent sequencing of the 16s rRNA gene and were amplified using universal primers, which included the 27-F forward primer and the 1492-R reverse primer. The protocol with certain modifications in line with the methodology described by Kumari et al. was followed in this study [30]. DNA isolation and amplification were performed on the isolates before assessing their probiotic potential. The quality of the obtained DNA was assessed using a UV spectrometer with nanodrop (Eppendorf, Bangalore). The PCR product amplified from the boza sample DNA was subjected to sequencing, followed by a partial sequence homology search using the Nucleotide BLAST program. The obtained partial sequences of DNA were submitted to the open-access GenBank database of NCBI, and accession numbers were assigned [30]. To create a phylogenetic tree, the 16S rRNA region of the two boza LAB isolates of boza from the current study was analysed using MEGA X (version 10.2.4) and a maximum likelihood phylogenetic tree with a 100 bootstrap consensus tree. The Tamura and Nei model [31] was found to be the most accurate in this study. The neighbor join algorithm and yet another algorithm called BioNJ were utilised to construct a pairwise distance matrix for generating the starting tree(s) in the heuristic search process [32].

### 2.3. Evaluation of Probiotic Attributes

#### 2.3.1. *In Vitro* Adhesion Assay

In this study, properties such as cell surface hydrophobicity using nonpolar solvent, autoaggregation (self-adhesion), and coaggregation (adhesion with pathogen) of isolated strains were assessed by following a modified procedure described by Abid et al. [33]. Autoaggregation was performed by incubating the 1 × 10^8^ CFU/mL of isolates in PBS (pH 6.8) for certain time intervals. To determine the coaggregation capability of the LAB isolates, a 2000 *μ*L bacterial suspension of the boza LAB isolates with a concentration of 1 × 108 CFU/mL was mixed with 4000 *μ*L of pathogenic strains obtained from MTCC such as ST-98, ML-1809, EC-443, PS-424, and BS-10403, and the mixture of the LAB isolate and pathogenic bacteria was incubated for about 2 h (37°C). At specific intervals, the difference in absorbance was measured to calculate the percentage of aggregation [34].

#### 2.3.2. *In Vitro* Adhesion Assay of Isolates to Epithelial Cells

RAMULAB29 and RAMULAB30 strains were assessed for their ability to adhere in vitro to human buccal lining epithelial cells and the HT-29 cell line derived from human colorectal cancer cells, serving as a model system. The approach by Kumari et al. [29] was used with a few minor modifications. Following a 30 min incubation period, Gram's staining was used for microscopic examination to determine whether LAB adhered to buccal epithelial cells. The HT-29 cell culture reached 70% confluence in the plate, followed by incubation with 1000 *μ*L of bacterial suspension (108 CFU/mL) for 60 min at 37°C. This facilitated the evaluation of bacterial adherence to the cells within 5% CO_2_ atmosphere. PBS was then added to remove nonadherent boza LAB isolates, from which the serial dilution was performed, and the nonadherent cells obtained were plated and incubated (37°C, 24 h). In order to assess the bacteria's adhesion ability, the CFU/mL ratio was calculated by comparing the initial boza LAB isolate cell number to the number of LAB isolate cells that remained after washing. This experiment was conducted in triplicate for each pair of samples.

#### 2.3.3. Bile Salt Tolerance and Acid Resistance

Tolerances to bile salts (using Ox Gall, SRL Pvt. Bangalore) under acidic conditions (similar to stomach pH) were assessed with a few minor modifications to the approach by Gomathi et al. (2014) [34]. The isolated LAB strains were introduced in two percentage variations (0.3 and 1%) in the MRS broth with a boza LAB isolate cell concentration of 10^8^ CFU/mL for two-hour intervals up to 4 h at a pH of 2 and a temperature of 37°C to determine the extent to which they tolerated acidic conditions and bile salts. A small volume of serial dilution (100 *μ*L) was plated at a specific time to determine the cell count, and after an incubation period of 24 h, the survival of the LAB isolate enumeration was observed.

#### 2.3.4. Assay for Simulated Gastric Juice Tolerance

To conduct the assay, we prepared simulated gastric juice to mimic stomach conditions and intestinal juice to simulate intestinal conditions. Pepsin (2500 U/mg, SRL Pvt. Ltd.TN, India) was dissolved in PBS with a pH of 3 at a concentration of 0.003 g/mL, while trypsin (2000 U/g, SRL Pvt. Ltd.TN, India) was dissolved in PBS with a pH of 8 at a concentration of 0.001 g/mL. Both solutions were then filter-sterilized by passing into a 0.22 *μ*m filter. Boza LAB isolates must be able to withstand digestion for up to 3 hours in stomach settings and up to 8 hours under intestinal conditions. The viable colony counts were utilised to determine the gastrointestinal tolerance of the chosen strain [35, 36].

### 2.4. Safety Evaluation

#### 2.4.1. Antibacterial Activity

The technique used to evaluate the antibacterial activity of boza-isolated LAB strains against pathogenic bacteria was performed using the agar well diffusion method [37]. KA-2822, BC-1272, SA-1144, BS-10403, KP-10309, ST-98, ML-1809, EC-443, PA-424, and BS-10403 were the test pathogenic organisms. These strains were chosen for assessment due to their relevance to food safety and public health concerns. First, plates were prepared, and the pathogenic bacteria were added and equally distributed throughout the agar surface. The wells were filled with 100 *μ*L of overnight-grown boza LAB isolates introduced into each well.

#### 2.4.2. Antibiotic Susceptibility

The susceptibility of boza LAB isolates was assessed by employing the disc diffusion method. LAB isolates were inoculated onto MRS agar plates at a concentration of 108 . Discs prefilled with antibiotics were then placed onto the plates and incubated (24 h, 37°C). The size of the clear zone of inhibition around each antibiotic disc was measured in millimetres (mm) to determine the susceptibility of boza LAB isolates to antibiotics. The antibiotic susceptibility pattern of the isolates was identified using the CLSI guidelines (2018) and was determined using antibiotic discs with defined concentrations per disc of streptomycin (0.1 mg), vancomycin (0.03 mg), tetracycline (0.03 mg), azithromycin (0.015 mg), methicillin (0.010 mg), and ampicillin (0.010 mg). The clear zone around the disc diameters of the discs was measured and interpreted according to the established performance criteria to classify the findings as susceptible, moderately susceptible, or resistant [38].

#### 2.4.3. Haemolytic Activity

The study by Hussain et al. [39] was referred to assess the haemolytic activity of the isolates, with a few slight modifications to the method. To conduct this test, streak plate inoculation was performed, and the agar plates containing sheep blood agar (w/v) were then incubated at 37°C for 48 hours. After incubation, the haemolytic (breakdown of RBC) activity of the boza isolates was determined by observing the lysis caused by RAMULAB29 and RAMULAB30 strains on red blood cells in the medium surrounding the colonies. The isolates were classified into three categories based on their haemolytic activity: *γ*-hemolysis, which is considered safe, *α*-hemolysis, and *β*-hemolysis.

### 2.5. Screening for Antioxidant Activity

The methodology described by Yadav et al. [40] was used to evaluate the isolates for ABTS scavenging of radicals, with some minor modifications. The absorbance measurement was taken at 750 nm. For the DPPH radical-scavenging capacity test, the method described by Sreepathi et al. [41] was used with slight modifications.

### 2.6. Carbohydrate-Hydrolysing Enzyme Inhibitory Assay

The cells were prepared as mentioned by Kumari et al. [29]. The method used to inhibit the AG enzyme involved the use of boza isolate extract samples, IC, CS, and CE, with slight modifications to the approach by Ademiluyi et al. [42]. To perform the AG enzyme inhibition assay, the boza isolate extract samples were first incubated in 0.05 M PBS buffer (pH 6.8) for 10 min, with a volume of 700 *μ*L. The sample combination was treated with the AG enzyme at a concentration of 0.25 U/ml, with a volume of 100 *μ*L, for 15 min. Subsequently, a substrate called pNPG was added to the mixture at a volume of 100 *μ*L, with a concentration of 5 mM. The reaction was allowed to continue for 30 min, after which it was stopped by adding 1,000 *μ*L of 0.1 M Na_2_CO_3_. For the enzyme AA inhibition assay, the protocol of Huligere et al. [43] was followed. First, the test sample was prepared with 500 *μ*L of sample and 500 *μ*L of 0.1 M PBS with a pH of 7.4. The AA enzyme was added to the sample at a concentration of 0.5 mg/mL and allowed to incubate for 10 minutes at 25°C. Following this, 500 *μ*L of 1% starch solution was added to the mixture and allowed to react for 10 min. Finally, the absorbance of the reaction of both enzymes AG (405 nm) and AA (540 nm) was measured using a microplate reader.

*X*_*S*_ refers to the absorbance of the sample and reactants, while *X*_*C*_ refers to the absorbance of the sample in the absence of reactants.

### 2.7. Statistical Analysis

The experiments in the present study were conducted in triplicate, and the results are presented as mean ± standard deviation. A statistical comparison of isolates was performed using an ANOVA and DMRT with the aid of SPSS software (version 21.0, Chicago, USA). The results were considered statistically significant if the *p* value was ≤0.05. GraphPad Prism version 8.0 software developed by GraphPad Software Inc was used to generate the graphs in this study.

## 3. Results

### 3.1. Identification and Characterization of LAB

The strains selected were rod shaped and characterized as Gram positive and catalase negative, resulting in the isolation of 10 strains accordingly (Supplementary Figure S2). In this study, the two isolates RAMULAB29 and RAMULAB30 were able to tolerate up to 4% NaCl and had the ability to withstand a pH of 2 to 6. The optimal temperature and pH for both the isolates were found to be 37°C and pH 7.4, respectively. Both strains demonstrated the ability to ferment sucrose glucose, lactose, and maltose articulated as heterofermentative strains (Table 1). The phenol tolerance assay led us to understand that 0.4% phenol is tolerable by isolates (Table 2).

### 3.2. Molecular Identification and Phylogenetic Assessment of LAB

**Figure 1:**
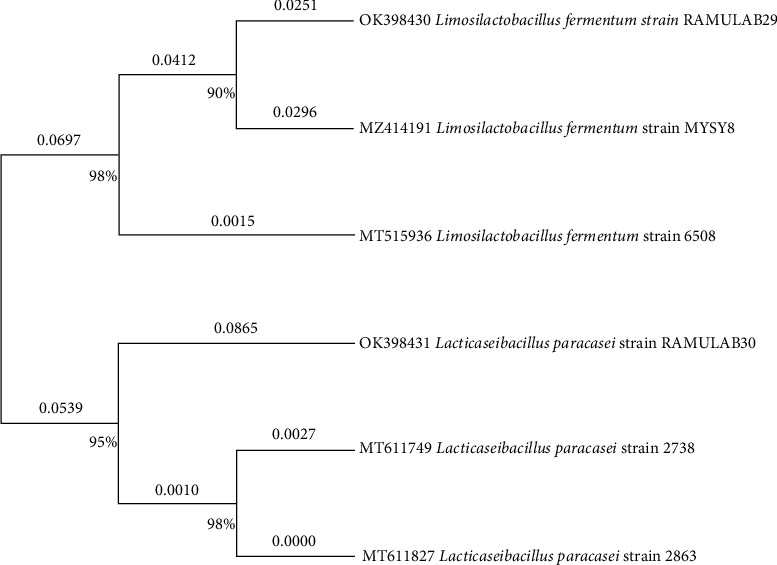
The phylogenetic tree provides information on the evolutionary relationships of RAMULAB29 and RAMULAB30 strains from boza samples based on maximum likelihood bootstrap analysis and provides information on the evolutionary history and relatedness of different bacterial strains based on their 16S rRNA sequences comparative with reference strains.

The DNA of RAMULAB28 and RAMULAB30 strain was PCR-amplified using 16s rRNA primers resulting in sequence lengths ranging from 1248 to 1424 bp. When comparing the sequences obtained with the sequences present in the GenBank database, it was observed that both the strains showed more than 95% similarity with the species *Limosilactobacillus fermentum* and *Lacticaseibacillus paracasei*. Furthermore, the NCBI GenBank Accession Number for RAMULAB28 and RAMULAB30 was identified as OK398430 and OK398431, respectively (Figure 1).

### 3.3. Probiotic Properties

#### 3.3.1. *In Vitro* Adhesion Assay

**Figure 2:**
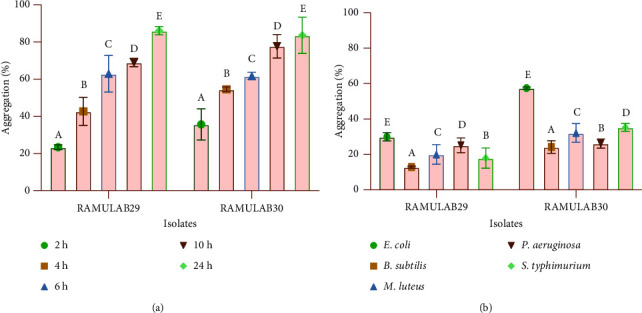
The autoaggregation and coaggregation data of the LAB strains were presented as mean ± SD. (a) Percentage of aggregation of RAMULAB29 and RAMULAB30 strains that autoaggregate over time at room temperature (28°C) and (b) aggregation percentage of RAMULAB29 and RAMULAB30 strains that coaggregate after 2 h at 28°C. DMRT was used to compare between means in the aggregation, and alphabetic superscripts (A–E) designate statistically significant differences (*p* < 0.05).

The hydrophobicity of the cells was assessed by utilising xylene. Specifically, Limosilactobacillus fermentum RAMULAB29 exhibited a survival rate of 65.71%,while Lacticaseibacillus paracasei RAMULAB30 showed a survival rate of 72.58% respectively (Table 2). Probiotic autoaggregation is required for bacterial colonization and protection. The degree of autoaggregation in the strains noticeably increased as the incubation time progressed. . This suggests that the cells had a greater tendency to bind to each other, resulting in larger and more tightly packed aggregates over time (Figure 2(a)). At 24 h, RAMULAB29 and RAMULAB30 strains had 86.09% and 83.64% autoaggregation capability, respectively. The coaggregation capacity of the two LAB isolates was evaluated with two indicator strains, EC-443 and BS-1272, and a control strain, ML-1809. The coaggregation results showed that the coaggregation capacity of both isolates was high with EC-443, indicating a strong interaction between the LAB strains and EC-443. However, the coaggregation ability was low with BS-1272, suggesting a weaker interaction between the LAB strains and BS-1272. ML-1809 showed a moderate coaggregation ability with both LAB isolates, indicating a moderate interaction between the two strains. Overall, the coaggregation capacity of LAB isolates was found to be strain-specific and depended on the indicator strain used in the assay, as shown in Figure 2(b).

#### 3.3.2. *In Vitro* Adhesion Assay of Epithelial Cells

According to the experimental findings, the isolates displayed the ability to attach to the buccal epithelial cells at a ratio of 80 to 100 bacterial cells/epithelial cells. The RAMULAB29 and RAMULAB30 isolates had the best ability to adhere to the epithelial cells (Supplementary Figure S3). Similar adhesions were observed with HT-29 cells; the isolates had adhesion higher than 75%, given in Table 2.

#### 3.3.3. Tolerance to Bile Salt under Acidic Conditions

**Figure 3:**
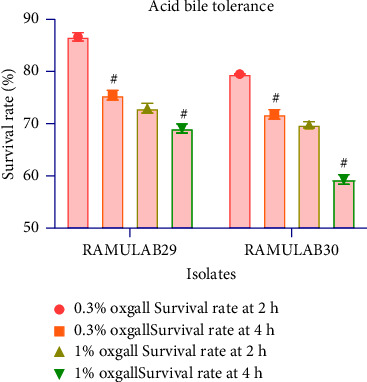
Survival rates of isolates at acidic pH 2 and different bile salt concentrations. The experiment was carried out by incubating the strains for 2 and 4 h (37°C) under 0.3% and 1% bile salt concentrations. Data are presented as mean ± SD, and means were compared using the DMRT with superscripts denoting significant differences (*p* < 0.05).

The determination of survivability at 0.3% and 1% oxgall concentrations in acidic environment (pH at 2) has shown that the isolates can tolerate this condition efficiently (Figure 3). The isolates have the ability to tolerate for 4 h and showed a survival of 71–75% at 0.3% oxgall concentration and 59–69% at 1% oxgall concentration. A reduction in survival rates up to 6% for RAMULAB29 and 12% for RAMULAB30 has been observed at 4 h of incubation. Hence, it was observed that the survival rate correlated with the bile concentration.

#### 3.3.4. Simulated Gastrointestinal Juice Tolerance Assay

**Figure 4:**
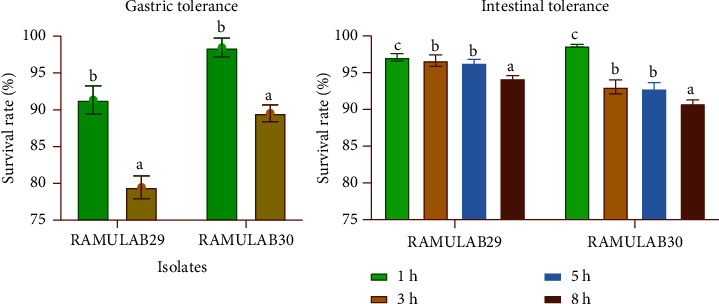
The results of the survival rates of the isolates in gastric and intestine juices are presented as mean ± SD. The means of the values obtained expressed the survival rates for different time intervals (1, 3, 5, and 8 h) and compared using DMRT, and the statistically significant differences were represented with different superscripts (a–c) (*p* < 0.05).

The isolates are considered being effective if they are capable of tolerating an extreme gastrointestinal environment. The results of the test indicated the potential of the isolates to withstand the acidic environment of the stomach and bile salts in the small intestine, suggesting their capacity for optimum growth and potential health benefits in the human gut. Both isolates were able to survive under this condition for a period of 8 h with little difference in the survival rate between isolates, as shown in Figure 4.

### 3.4. Safety Assessments

#### 3.4.1. Antibacterial Activity

The ability of RAMULAB29 and RAMULAB30 strains to inhibit the growth of pathogenic bacteria was investigated. The results showed that, with the exception of ST-98 and KA-2822, the isolates were capable of inhibiting all the tested pathogens, as indicated by the zone of inhibition ranging from 6 to 16 mm. ML-1809 was the pathogen most sensitive to both isolates, while the least sensitive was BC-1272 (Table 3).

#### 3.4.2. Antibiotic Sensitivity

The susceptibility or resistance of the two isolates was determined by subjecting them to six different antibiotics. The results showed that RAMULAB29 and RAMULAB30 strains were resistant to vancomycin (V) and also methicillin (MET), but the higher susceptible result was obtained for streptomycin (STR) and ampicillin (AMP), while RAMULAB28 and RAMULAB30 showed moderate susceptible to tetracycline (TET) and azithromycin (AZM). The reference standard chart was used for comparison to obtain these results (Table 4).

#### 3.4.3. Haemolytic Assay

There is an absence or lack of a distinct or well-defined zone surrounding the colonies, known as *γ*-hemolysis, which indicates that the organism is nonhaemolytic and safe. Isolates RAMULAB29 and RAMULAB30 did not exhibit any haemolytic activity, as observed by the absence of a clear zone with no lysis of RBC around the colonies. This suggests that the isolates did not cause hemolysis.

### 3.5. Antioxidant Assay

**Figure 5:**
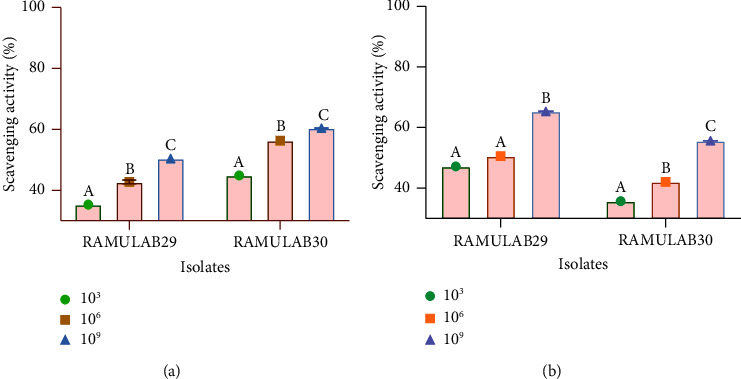
Results of the isolate's scavenging activity of DPPH and ABTS radicals. (a) ABTS scavenging activity and (b) DPPH scavenging activity. Data are expressed as mean ± SD. DMRT was used to compare the mean ± SD of the scavenging activity at various CFU/ml, and those with distinct superscripts (A–C) are significantly different (*p* < 0.05).

The experiment carried out showed that as the cell count (measured in CFU/ml) of RAMULAB29 and RAMULAB30 strains increased, their scavenging activity for ABTS and DPPH also increased. Upon testing at a concentration of 10^9^ CFU/mL, RAMULAB29 and RAMULAB30 strains demonstrated the ABTS scavenging activity of 50.43% and 60.42%, respectively. Similarly, the DPPH scavenging activity at 10^9^ CFU/ml was found to be 65.30% for RAMULAB29 and 55.62% for RAMULAB30 (Figure 5).

### 3.6. Carbohydrate Hydrolysing Enzymes' Inhibitory Assay

**Figure 6:**
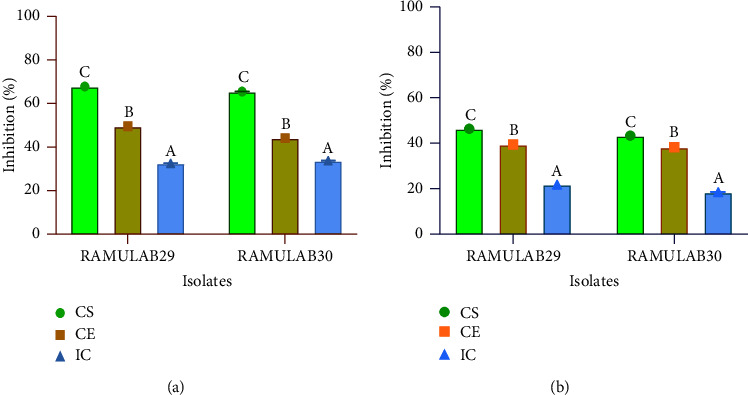
The inhibitory activity of the two isolates against the enzymes AG and AA was tested using IC, C, and CE. The results showed the isolate extract's ability to hinder the enzymes AG (a) and AA (b). Data are presented as mean ± SD, and the DMRT was used to determine the significance of means of the inhibitory activity of the isolate extracts with different alphabetic superscripts (A–C) indicating a significant difference (*p* < 0.05).

The study carried out tests to evaluate the inhibitory activity of two isolates against the enzymes AG and AA, using IC, CS, and CE. When tested with CS, the isolates demonstrated a stronger inhibitory potential than with IC and CE. Both RAMULAB289 and RAMULAB30 demonstrated the inhibitory activity against the AG enzyme ranging from 32 to 67%. The inhibition of AA by each isolate ranged from 18 to 46% (Figure 6).

## 4. Discussion

*Lactobacillus* spp. has consistently been used in food fermentation, and its use has spawned a new field and discipline of functional foods. Throughout human history, fermented foods have played a significant role in our diets, and the fermentation process has long been linked to a range of health benefits dating back to ancient times [44]. The main objective of this present investigation was to discover and determine boza probiotic bacteria that have the ability to endure the process of digestion and prevent the activity of carbohydrate-hydrolysing enzymes, specifically AG and AA. Fermented foods have a higher nutritional content, functional qualities, and digestibility than nonfermented foods. In the preparation of boza, with minor variations in the production techniques, cereals are always the key ingredient. In addition to carbohydrates, grains contain minerals, sterols, and vitamins that aid in the development of microbes [45]. According to previous studies, LAB such as *Leuconostoc* spp., *Lactobacillus* spp., and Enterococcus spp. were isolated from boza [23, 46, 47]. In our study, the strains of *Lacticaseibacillus paracasei* and *Limosilactobacillus fermentum* are isolated from the fermented boza sample.

The capability of the isolate to tolerate harsh conditions in the gut is a remarkable ability of probiotics. According to the assays, the strains were found to have the ideal growth temperature of 37°C and the optimal pH of 7.4, with the tolerance ability up to 4%. These are the minimum requirements of the probiotic potential LAB. Furthermore, phenol is a bacteriostatic substance formed in the stomach as a result of gut microorganisms that deaminate aromatic amino acids [48]. Both isolates also showed phenol tolerance (0.4%), and a viable rate was observed greater than 95% (24 h). In addition to favourable growth conditions, it is crucial for the isolates to exhibit resistance to digestion and survive the hostile conditions of the gastric tract, which is the stomach, for up to 3 hours and the intestinal environment for 3 to 8 hours, where the pH levels can vary highly with acidic conditions of pH 2 in the stomach to more alkaline levels of pH 8 in the intestine. This characteristic is crucial for probiotics as they must be able to survive and reach the intestines to exert their health benefits [49]. *L. plantarum* L7 isolated from the rice-based fermented beverage expressed an almost 50% reduction in tolerance to the survival rate for the 0.3% bile at acidic pH of 2 [50]. In this study, the RAMULAB29 and RAMULAB30 strains both had a survival rate of over 70% for 0.3% bile concentration (pH 2), wherein the survival rate decreased by 10% as the bile concentration increased to 1% (pH 2), indicating that the isolated strains are capable of tolerating 0.3% bile conditions. Huligere et al. [51] demonstrated that the survival rate of the potential probiotic *P. pentosaceus* VJ13 decreased by 20% after exposure to gastric juice for 4 hours, with a decrease in the cell numbers from 9.11 to 6.58 CFU/mL. Similarly, exposure to intestinal juice resulted in a reduction in the cell numbers from 9.29 to 8.17 CFU/ml. RAMULAB29 and RAMULAB30 strains had a survival rate greater than 75% for gastric juice and greater than 90% for intestinal tolerance at 3 h, with little variations observed in the survival rate at 1 h of incubation. The enzymes of the gastric juice such as pepsin are likely to damage the cells, causing a rupture resulting in low survival at the gastric pH of 2. However, the strains identified in the current study demonstrated resistance, possibly due to their ability to withstand such harsh digestion conditions.

Probiotics can help inhibit pathogen colonization due to their hydrophobicity, autoaggregation, and coaggregation characteristics. In the case of bacteria from the same genus, they may share similar autoaggregation and hydrophobicity properties, which can facilitate their adhesion to the gut layer. This adhesion can provide several benefits, such as improved colonization, enhanced survival under harsh environmental conditions, and increased competition with other microorganisms for resources. Furthermore, adhesion to the gut layer can also facilitate the delivery of beneficial compounds or proteins produced by bacteria, which can have a positive impact on the host health [52, 53]. Several previous investigations have revealed strain-dependent adhesion with hydrocarbons, which is consistent with our findings [54–56]. Binding to hydrophobic surfaces has been shown to be a time-independent interaction that involves several tiny length forces of unfolded microbial cell surface proteins [57]. Microbial adhesion is a complex process that involves interactions between bacterial surfaces and host tissues. Electrostatic interactions and hydrophobic interactions are two mechanisms involved in this process. Electrostatic interactions occur because of differences in charges between the bacterial cell surface and host tissue, while hydrophobic interactions involve short-range forces such as van der Waals forces and hydrogen bonding. These interactions promote the adhesion of bacterial cells to host tissue, which can be important for the probiotic activity in the gut [58]. The study by Li et al. aimed to investigate the probiotic properties of *Lactobacillus* salivarius M2-71 by evaluating its autoaggregation and coaggregation abilities with *Escherichia coli*. Their results showed that *L. salivarius* M2-71 exhibited a gradual increase in autoaggregation over time and a corresponding increase in coaggregation with *E. coli*, showing the potential probiotic activity. The results obtained in the current study align with previous findings and provide additional evidence supporting the potential probiotic properties of the isolated strains [53]. This occurrence contributes to the intestine's ability to maintain a healthy environment. The isolates are probiotics that are generally recognised as safe (GRAS). However, putative probiotics should undergo basic safety tests, which includes evaluating their antibiotic resistance patterns. It is important to ensure that the strains do not pose a risk to human health and that they can be used safely as probiotics [59, 60]. Antibiotic resistance testing can help identify potential risks associated with the use of probiotics and ensure that strains selected for use as probiotics do not pose the risk of spreading antibiotic resistance to pathogenic bacteria [52]. *Limosilactobacillus fermentum* RAMULAB28 and *Lacticaseibacillus paracasei* RAMULAB30 isolated in the present study were resistant to vancomycin and methicillin. The generated zone, which is based on published guidelines for the interpretation of zone sizes in the CLSI 2018 scale, specifies how the resistance and sensitivity profile should be interpreted [38]. The antibacterial effect of probiotic strains against infections is another important factor in overall health and well-being, which can be significantly impacted by maintaining a healthy microflora balance in the intestine. Likewise, for centuries, fermentation has been used to preserve food due to the production of antimicrobial metabolites by microorganisms during this process. Metabolites such as organic acids, alcohols, and bacteriocins create acidic and alcoholic environments that are unfavourable for the growth of pathogenic microorganisms. As a result, fermented foods have a longer shelf lifetime and fewer are likely to be contaminated, making them a safe and healthy option for consumption [61]. Probiotics are known to employ various mechanisms to exert their beneficial effects in the host's gut, including the production of antimicrobial compounds [62]. Usually, *Lactobacilli* spp. can potentially generate antimicrobial compounds including lactic acid, which is produced during carbohydrate fermentation and can lower the pH of the gut, creating an unfavourable environment for pathogenic microorganisms and superoxide radicals, which are highly reactive molecules, which can damage bacterial cell walls and membranes, leading to cell death, and/or antimicrobial peptides such as bacteriocins, which are small peptides that have an antimicrobial activity against a range of pathogens, including bacteria, yeasts, and fungi, which add to their probiotic effects [63, 64]. In this study, the RAMULAB29 and RAMULAB30 isolates had the highest inhibition zone against *M. luteus* MTCC-1809, but the least inhibition against *B. cereus* MTCC-1272. Probiotic bacteria are said to mediate displacement or inhibition mostly by producing antimicrobial metabolites or antiadhesive factors rather than competing for common adhesion sites [65]. When it comes to probiotic safety, the absence of the haemolytic activity is critical. Both isolates in this study had no haemolytic activity, and similar results were obtained in many studies [66, 67]. As a result, the strains are not virulent and lacking in haemolysin [56]. In addition to the investigated features related to probiotic properties, it is also interesting to evaluate functional characteristics of *Lactobacillus* spp., such as their antioxidant capacity. An imbalance in the production of reactive oxygen species (ROS) can lead to oxidative stress, compromising the host's antioxidant defense system. ROS scavenging and cellular protection from oxidative damage are some of the key benefits of antioxidants, which are specialized compounds with this unique ability. Therefore, the ability of probiotics to act as antioxidants has the potential to confer a variety of health benefits on the host [68]. When antioxidants react with these free radicals (DPPH and ABTS), they take electrons or hydrogen atoms from them and become irreversibly stable [68]. The antioxidant activity of *Lactobacillus* spp. significantly contributes to the prevention of various diseases, including heart disease, gastrointestinal disorders, and diabetes [69, 70]. The results obtained were similar to those reported by Kim et al. [68] for *Lactobacillus* spp., which also showed a high antioxidant activity against these assays at a concentration of 109 CFU/g. The study by Muccee et al. suggested that modifying the catalytic sites of GUS enzymes could be a potential strategy for targeting breast cancer tumors. GUS enzymes have been found to enhance the estrogen concentration in the blood through the deconjugation of glucuronidated estrogens [71]. In the study by Akmal et al., PCR140 (NMCC91), belonging to the genus *Lactobacillus*, from homemade pickles demonstrated the highest in vitro probiotic and antioxidant potential [72]. Overall, the study aimed to provide information on the potential health benefits of isolated probiotic strains.

This study primarily aimed to evaluate the effectiveness of the probiotic strains that were extracted from boza in suppressing carbohydrate-hydrolysing enzymes such as AG and AA. Inhibiting intestinal AG is a widely recognised method for managing postprandial hyperglycemia [11]. Despite the fact that enzyme AG inhibitors are frequently used in clinical research, alternative enzyme AG inhibitors are being researched all the time with the aim of reducing side effects and drug costs. Functional foods with the inhibitory activity have also been investigated, and a variety of plants or food, rather than synthetic or artificial sources, have been investigated [37, 73]. Another enzyme widely studied for its inhibition is the *α*-amylase, an enzyme involved in the breakdown of starch and glycogen. It is being studied as a therapy for carbohydrate uptake conditions such as diabetes and obesity [63]. The enzymes AA and AG are responsible for hydrolysing complex carbohydrates, such as oligosaccharides and disaccharides, into monosaccharides that can be absorbed and metabolised by the body. These enzymes slow the process of digestion and prolong the time it takes for carbohydrates to be digested. As a consequence, the rate of glucose absorption slows, leading to a reduction in postprandial plasma glucose levels. Slowing the digestion process and prolonging carbohydrate digestion time can be achieved by inhibiting these enzymes. Inhibition of these enzymes leads to a reduction in the rate at which glucose is absorbed, resulting in lower levels of postprandial plasma glucose [74, 75]. *Lactobacillus* spp. has been explored from various sources to investigate the inhibition of carbohydrate-hydrolysing enzymes [63, 76]. In addition to this, the probiotic ability of the LAB facilitates them in releasing certain factors into the media that work similarly to the plant bioactive with a more potent ability without affecting the bacteria itself physiologically. With this perspective, in the inhibitory activity of the previous study, enzyme AA for crude and refined extracts of exopolysaccharide produced from Lactiplantibacillus plantarum H31 varied. Likewise, in our study, inhibition varied among CS, IC, and CE [77]. In that, RAMULAB29 and RAMULAB30 strains expressed a higher inhibition exerted by CS as analogous to the study by Son et al. [78] where L. brevis strain expressed a higher inhibitory effect for a cell-free supernatant. Kim et al. (2018) [79] evaluated the *L. plantarum* strain isolated from homemade kimchi, which had the competence to inhibit both enzymes with an inhibition greater than 95%. Zhong et al. [80] assessed effects of different types of fermentation on their potential inhibitory efficiency against carbohydrate hydrolysis. This study evaluated fermentation methods including fermentation on its own, commercial starter fermentation, and spontaneous fermentation. When compared to natural fermentation, the inhibition of enzymes AG and AA was higher in self- and commercial starter fermentation. In the present study, boza fermentation occurred naturally with a promising ability to inhibit both enzymes AG and AA.

## 5. Conclusion

In conclusion, this is the first investigation to indicate that boza is capable of demonstrating its ability to harbour putative probiotic property. Specifically, the strains RAMULAB29 and RAMULAB30 exhibited remarkable probiotic traits and displayed antioxidant capabilities by scavenging DPPH and ABTS radicals. Furthermore, the strains *Lacticaseibacillus paracasei* RAMULAB30 and *Limosilactobacillus fermentum* RAMULAB29 showed promising inhibitory effects against *α*-glucosidase and *α*-amylase enzymes. The findings highlight the significance of boza as a potential source of probiotic LAB isolates for combating diabetes. These identified strains hold promise for further research and development in the field of probiotics and diabetes management, offering potential benefits for intestinal health and antidiabetic interventions.

## Figures and Tables

**Table 1:** The phenotypic traits and fermentation capacity of LAB strains isolated from boza sample.

Tests	Isolates^*∗*^	
	RAMULAB29	RAMULAB30
Gram staining	P	P

Catalase	N	N

Morphology	R	R

*Occurrence of growth at different temperatures (°C)*		
4	N	N
10	N	N
37	P	P
45	N	N
50	N	N

*Growth tolerance in varying NaCl concentrations (%)*		
2.0	P	P
4.0	P	P
7.0	N	N
10.0	N	N

*Carbohydrate fermentation*		
Glucose (C_6_H_12_O_6_)	P	P
Xylose (D) (C_5_H_10_O_5_)	N	N
Xylose (L) (C_5_H_10_O_5_)	N	N
Sucrose (C_12_H_22_O_11_)	P	P
Mannitol (C_6_H_14_O_6_)	N	N
Maltose (C_12_H_22_O_11_)	P	P
Lactose (C_12_H_22_O_11_)	P	P
Galactose (C_6_H_12_O_6_)	N	N
Arabinose (C_5_H_10_O_5_)	N	N
Starch (C_6_H_10_O_5_)n	N	N

*Growth tolerance at different pH values*		
2	P	P
4	P	P
6	P	P
7.4	P	P

^*∗*^“P” indicates presence, “N” indicates absence, and “R” indicates rod.

**Table 2:** The boza sample isolates' phenol tolerance, cell surface hydrophobicity, and adhesion of HT-29 cells.

Isolates	Phenol tolerance (log CFU/mL)^*∗*^		Cell surface hydrophobicity (%)^*∗*^	HT-29 adhesion (%)^*∗*^
	0 h	24 h		
RAMULAB29	7.42 ± 0.08^b^	7.30 ± 0.08^a^	65.71 ± 3.91^a^	78.34 ± 0.12^a^
RAMULAB30	7.38 ± 0.03^a^	7.64 ± 0.55^b^	72.58 ± 6.06^b^	89.86 ± 0.59^b^

^*∗*^The mean values of the results are accessible as mean ± SD. To determine significant differences between means, DMRT was applied, and means (column) that were marked with different alphabetic letters (a, b) were considered statistically significant differences (*p* ≤ 0.05).

**Table 3:** Antibacterial activity of RAMULAB29 and RAMULAB30 strains.

Isolates		RAMULAB29	RAMULAB30
Pathogens	KP-10309	+	+
	EC-443	++	++
	BC-1272	+	+
	BS-1272	+	+
	KA-2822	−	−
	ML-1809	+++	+++
	PA-424	++	+++
	PF-667	++	++
	SA-1144	++	++
	ST-98	−	−

^*∗*^Zones of inhibition are indicated in millimetres (mm) with the following symbols: (−) for no inhibition, (+) for minimal inhibition with a zone size of 5 mm, (++) for adequate inhibition with a zone size greater than 6 mm, and (+++) for robust inhibition with a zone size greater than 16 mm.

**Table 4:** Antibiotic susceptibility test of the RAMULAB29 and RAMULAB30 strains representing resistance and sensitivity based on CLSI, 2018 [38].

Sl. no.	Antibiotic	The inhibitory zone (S/R mm)	RAMULAB29	RAMULAB30
1	STR	(≥15/≤12)	S	S
2	V	(≥17/≤14)	R	R
3	TET	(≥19/≤14)	S	S
4	AZM	(≥13/≤12)	S	S
5	AMP	(≥17/≤14)	S	S
6	MET	(≥22/≤17)	R	R

V, STR, TET, AZM, AMP, and MET. The area of inhibition (measured in millimetres) caused by the suitable antibiotics is where the sensitivity or resistance (S/R) breakpoints are indicated.

## Data Availability

The datasets presented in this study can be found in online repositories. The names of the repository/repositories and accession number(s) can be found in the article.

## Abbreviations

AG: *α*-glucosidase
AA: *α*-amylase
GRAS: General recognition as safe
CS: Supernatant of the isolate
CE: Lysed extract of the isolate
IC: Intact cell of the isolate
FAO: Food and Agriculture Organization of the United Nations
WHO: World Health Organization
LAB: Lactic acid bacteria
BLAST: Basic local alignment search tool
NCBI: National Center for Biotechnology Information, National Institutes of Health, USA
DNA: Deoxyribonucleic acid
CFU/mL: Colony-forming units per milliliter
16S rRNA: 16S ribosomal RNA
MTCC: Microbial Type Culture Collection and Gene Bank, Chandigarh, India
ST-98: Salmonella typhimurium-98
ML-1809: Micrococcus luteus-1809
EC-443: Escherichia coli-443
PS-424: Pseudomonas aeruginosa-424
BS-10403: Bacillus subtilis-10403
KA-2822: K. aerogenes-2822
BC-1272: Bacillus cereus-1272
SA-1144: Staphylococcus aureus-1144
KP-10309: Klebsiella pneumonia-10309
RBC: Red blood cell
Na_2_CO_3_: Sodium carbonate
DMRT: Duncan's multiple range test
ANOVA: Analysis of variance
SPSS software: Statistical Package for the Social Sciences Software
ABTS: 2,2′-azino-bis(3-ethylbenzothiazoline-6-sulfonic acid)
DPPH: 2,2-diphenyl-1-picrylhydrazyl
PBS buffer: Potassium phosphate buffer
CLSI: Clinical and Laboratory Standards Institute
pNPG: p-nitrophenyl-D-glucopyranoside.

## Supplementary Materials

Supplementary Materials The supplementary materials include additional figures and tables that complement and support the main content of the study. Supplementary Figure S1: The process of making homemade boza. Supplementary Table S1: Ingredients used for the preparation of boza. Supplementary Figure 2: MRS agar plates showing distinct colonies of *Lactobacillus* spp. and microscopic view (100X) of Gram's-stain isolates (A) RAMULAB29 and (B) RAMULAB30. Supplementary Figure S3: Adherence of the boza LAB strain to buccal lining epithelial cells was observed using a light microscope: Figure (B) shows the adhesion of RAMULAB29 and RAMULAB30 isolates to buccal lining epithelial cells, while Figure (A) serves as the control. Attached isolates are indicated by an arrow.
