# Supplementary material for: Characterization of Lactobacillus spp. as Probiotic and Antidiabetic Potential Isolated from Boza, Traditional Fermented Beverage in Turkey
Source: Int J Microbiol. 2024 Jun 10;2024:2148676. doi: 10.1155/2024/2148676 (PMC11221989; doi:10.1155/2024/2148676)
Supplement: Supplementary Materials — The supplementary materials include additional figures and tables that complement and support the main content of the study. Supplementary Figure S1: The process of making homemade boza. Supplementary Table S1: Ingredients used for the preparation of boza. Supplementary Figure 2: MRS agar plates showing distinct colonies of Lactobacillus spp. and microscopic view (100X) of Gram's-stain isolates (A) RAMULAB29 and (B) RAMULAB30. Supplementary Figure S3: Adherence of the boza LAB strain to buccal lining epithelial cells was observed using a light microscope: Figure (B) shows the adhesion of RAMULAB29 and RAMULAB30 isolates to buccal lining epithelial cells, while Figure (A) serves as the control. Attached isolates are indicated by an arrow. [file 2148676.f1.docx]

**Characterization of *Lactobacillus* spp. as probiotic and antidiabetic potential isolated from Boza, Traditional Fermented Beverage in Turkey**

**Chandana Kumari V B^1^, Sujay Huligere^1^,** [**Jayanthi**](https://pubmed.ncbi.nlm.nih.gov/?term=Gangadharappa+BS&cauthor_id=36480100) **M K^2^, Khang Wen Goh^3^, Sudhanva M. Desai^4^, Kalabharthi H L^2*^, and Ramith Ramu^1*^**

*^1^Department of Biotechnology and Bioinformatics, JSS Academy of Higher Education and Research, Mysore – 570015, Karnataka, INDIA* [*chandanavb2@gmail.com*](mailto:chandanavb2@gmail.com) *(C.K.V.B.);* [*sujayhuligere@gmail.com*](mailto:sujayhuligere@gmail.com) *(S.S.H.)*

*^2^Department of Pharmacology, JSS Medical College, JSS Academy of Higher Education and Research, Mysore – 570015, Karnataka, INDIA* [*mkjayanthi@jssuni.edu.in*](mailto:mkjayanthi@jssuni.edu.in) *(J.M.K.);* [*drkala14@gmail.com*](mailto:drkala14@gmail.com) *(K.H.L.)*

***^3^****Faculty of Data Science and Information Technology, INTI International University, Nilai 71800, Malaysia* [*khangwen.goh@newinti.edu.my*](mailto:khangwen.goh@newinti.edu.my) *(K.W.G.)*

***^4^*** *Department of Chemical Engineering, Dayananda Sagar College of Engineering, Bengaluru, Karnataka, India* [*desai-chml@dayanandasagar.edu*](mailto:desai-chml@dayanandasagar.edu) *(S.M.D.)*

********Correspondence:* [*ramith.gowda@gmail.com*](mailto:ramith.gowda@gmail.com)*;* [*drkala14@gmail.com*](mailto:drkala14@gmail.com)


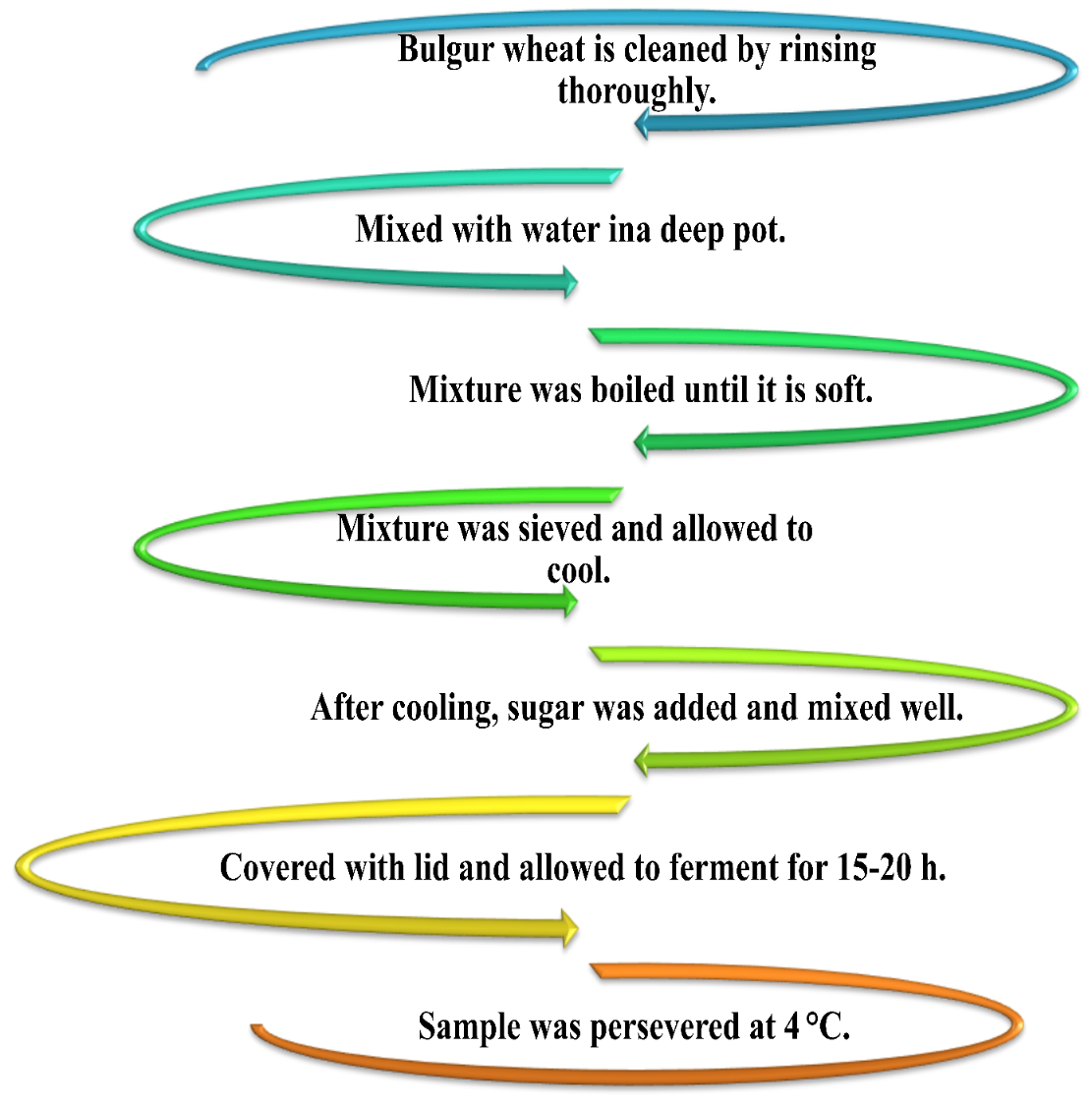


**Supplementary** **Figure S1.** The process of making homemade boza.

**Supplementary** **Table S1.** Ingredients used for the preparation of boza.

| **Ingredients** | **Quantity** |
| --- | --- |
| Bulgur wheat | 1 and ½ cups |
| Water | 10 cups |
| Sugar | 2-3 table spoons |


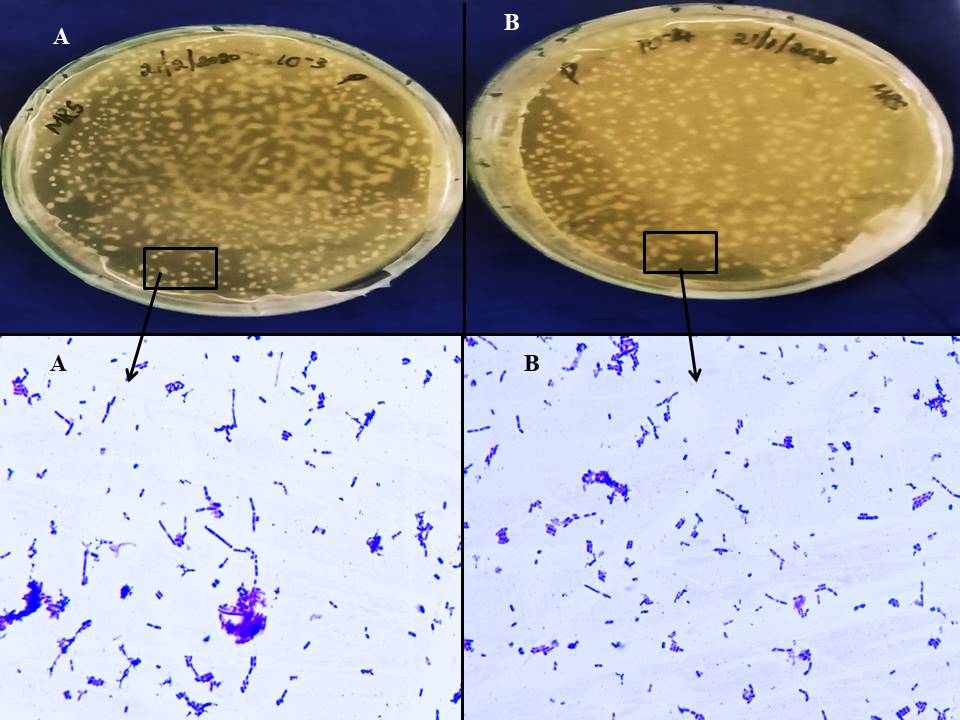


**Supplementary Figure S2.** MRS agar plates showing distinct colonies of *Lactobacillus* spp. and microscopic view (100X) of Gram’s-stain isolates (A) RAMULAB29, (B) RAMULAB30.


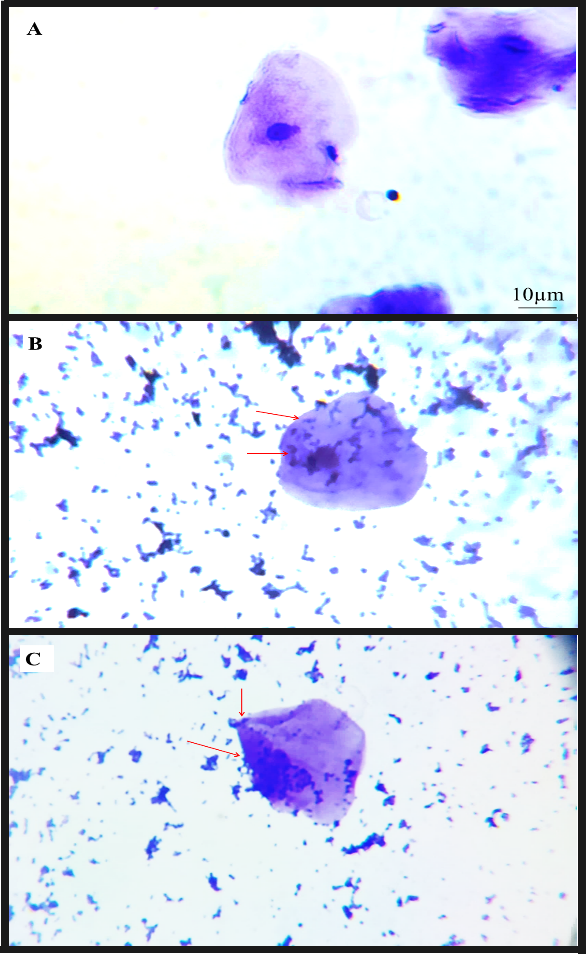


**Supplementary Figure S3.** Adherence of the Boza LAB strain to buccal lining epithelial cells was observed using a light microscope. Figure (B) shows the adhesion of RAMULAB29 and RAMULAB30 isolates to buccal lining epithelial cells, while Figure (A) serves as the control. Attached isolates are indicated by an arrow.
